# Long-term survival and success rates of immature versus root-end resected mature tooth autotransplants: part I of a retrospective cohort study

**DOI:** 10.1007/s00784-026-06990-w

**Published:** 2026-07-07

**Authors:** Tobias Zeller, Emilio Couso-Queiruga, Simone F. M. Janner, Michael M. Bornstein, Valerie G. A. Suter, Vivianne Chappuis, Clemens Raabe

**Affiliations:** 1 Department of Oral Surgery and Stomatology, School of Dental Medicine, University of Bern, Freiburgstrasse 7, Bern, CH-3010 Switzerland; 2 Clinic of Oral Surgery, University Center for Dental Medicine Basel UZB, University of Basel, Basel, Switzerland; 3 Surgeon, Surgery Center ZIKO Bern, Bern, Switzerland; 4 Department of Oral Health & Medicine, University Center for Dental Medicine Basel UZB, University of Basel, Basel, Switzerland

**Keywords:** Autotransplantation, Apicoectomy, Root-end resection, Revascularization

## Abstract

**Objectives:**

To compare the survival and success rates of autotransplanted immature teeth versus mature teeth treated with extraoral root-end resection (EORER), and assess inter-rater agreement in radiographic evaluation between oral surgeons (OS) and general practitioners (GPs).

**Materials and methods:**

Patients who underwent autotransplantation between 2000 and 2022 were retrospectively identified and invited for clinical and radiographic follow-up examinations. Periapical radiographs were independently evaluated by three OS and three GPs. Predictors of survival and success were analyzed using univariate generalized estimating equation models.

**Results:**

Fifty-eight autotransplanted teeth (39 immature, 19 mature) in 50 patients were included. Immature transplants showed higher survival (89.7% vs. 73.7%; mean follow-up 8.4 vs. 5.6 years) and higher success rates (63.3% vs. 31.3%) than EORER-treated mature transplants. Pulp revascularization was observed in 88.5% of immature and 54.5% of EORER-treated mature teeth. Longer splinting and pristine recipient sites were associated with higher survival; premolar and molar sites with higher success (p **≤** 0.047). Inter-rater agreement was lowest for pulp canal obliteration, with OS showing higher agreement than GPs (*p* = 0.02). GPs generally reported more pathological findings on the radiographs.

**Conclusions:**

Immature transplants demonstrated higher survival and success rates than EORER-treated mature transplants. However, pulp revascularization was observed in over half of mature transplants. Radiographic assessment varied considerably between OS and GPs.

**Clinical Relevance:**

Immature donor teeth remain the preferred option for autotransplantation. Nevertheless, EORER extends eligibility to mature donor teeth by promoting pulp revascularization, potentially avoiding root canal treatment. Standardized radiographic criteria and calibration are warranted for follow-up over time.

**Supplementary Information:**

The online version contains supplementary material available at 10.1007/s00784-026-06990-w.

## Introduction

Tooth autotransplantation is a biologically driven option for replacing missing teeth, particularly in children and young adults. Unlike dental implant therapy, it may re-establish a functional periodontium, supporting continued development of the surrounding hard and soft tissues, permitting orthodontic movement, providing proprioceptive function, and potentially offering a more cost-effective long-term solution [1–5]. Its application is nevertheless constrained by strict case selection, a suitably positioned donor tooth, favorable hard- and soft-tissue conditions at the recipient site, surgical routine and efficiency, and patient compliance [6, 7]. Consequently, tooth autotransplantation is still a comparatively rare procedure, typically performed in specialist centers.

Successful clinical and radiographic outcomes following tooth autotransplantation depend on two biologically distinct healing processes that may fail independently: periodontal and pulpal healing. Periodontal healing can be jeopardized by damage to the root cementum during donor-tooth extraction and extraoral handling, which may result in ankylosis or replacement resorption [8, 9]. Pulpal healing depends on revascularization across the apical foramen and failure of this process might lead to pulp necrosis, infection and inflammatory root resorption [9, 10]. Both healing pathways are closely related to the stage of root development at the time of surgery [11]. Immature teeth, with open apices and a developing periodontal ligament, achieve revascularization rates exceeding 90% alongside favorable periodontal healing [12–16]. In contrast, mature teeth with complete root and apex formation, by contrast, show spontaneous pulpal healing in only about 16% of cases and are routinely subjected to prophylactic root canal treatment and exhibit root resorptions or ankylosis more frequently [15–18].

To promote pulp revascularization in patients with fully developed mature donor teeth, extraoral root-end resection (EORER) has been proposed as a means of removing the apical constriction, enlarging the pulp surface exposed to the recipient bed, and potentially obviating prophylactic endodontic treatment [19]. Two retrospective studies have reported survival rates of 90–96.5% and pulpal healing in 55.6–94.7% of mature transplants managed with EORER, markedly exceeding revascularization rates of unmodified mature transplants [15, 20, 21]. However, medium to long-term data remain scarce, and direct comparisons with conventional immature-tooth autotransplantation within the same cohort are lacking.

Beyond the surgical procedure itself, accurate radiographic follow-up is essential, as autotransplanted teeth frequently develop sequelae such as pulp canal obliteration, apical periodontitis, and inflammatory or replacement resorption that may be radiographically apparent before clinical signs emerge and may prompt timely intervention [22, 23]. Yet most autotransplants are monitored in general practice rather than in specialist centers, and the extent to which clinician experience and background influence radiographic interpretation of this uncommon treatment has not been investigated.

The present study is Part I of a two-part retrospective cohort study and primarily aimed to compare long-term survival and success of autotransplanted immature teeth with those of mature teeth treated with EORER. Secondary aims included (1) the identification of potential predictors of survival and success; (2) to characterize clinical findings, including pulp revascularization at follow-up; and (3) to compare radiographic image interpretation of autotransplanted teeth between oral surgeons (OS) and general dental practitioners (GP).

## Materials and methods

This retrospective study took place at the Department of Oral Surgery and Stomatology, University of Bern, Switzerland, between November 2023 and September 2025. Ethical approval was granted by the Ethics Commission of the State of Bern (KEK-BE: 2023 − 01338, Bern, Switzerland). The study was conducted in accordance with the principles of the Declaration of Helsinki, as revised in 2024, and followed the STROBE (Strengthening the Reporting of Observational Studies in Epidemiology) guidelines [24]. All participants provided written informed consent prior to inclusion in the study.

### Patient selection and surgical protocol

Patients who underwent tooth autotransplantation at the Department of Oral Surgery and Stomatology, University of Bern, between 2000 and 2022 were identified through an electronic search of the local clinical database. Eligible patients were contacted by telephone and invited to attend a one-time follow-up examination. A minimum postoperative period of 12 months was required for inclusion. Exclusion criteria were pregnancy at the time of follow-up, missing records, and refusal to participate. When patients declined in-person follow-up, information on the transplant status, specifically whether the tooth remained in situ and whether any pain or discomfort was present, was collected during the phone call. Information on surgical variables was retrieved from patient charts and existing radiographs. Teeth were classified as immature if root formation was incomplete with an open apex (Moorrees stages R¼ to Rc), and as mature if an apical constriction had formed (stages A½ and Ac) [25].

All surgeries were performed under local anesthesia by senior OS. Recipient sites were prepared by extracting a hopeless tooth when present or, at edentulous sites with a pristine alveolar ridge, by raising a mucoperiosteal flap and preparing an osteotomy approximately 1–2 mm larger than the donor tooth’s dimensions. Following minimally traumatic extraction, the donor tooth was stored in a cell-preserving medium (SOS Dentobox, Miradent, Duisburg, Germany; or Dentosafe, Kaladent AG, St. Gallen, Switzerland). For mature donor teeth, an EORER of 3–4 mm was performed with a sterile fissure bur under cooled irrigation. The donor tooth was then seated in the recipient site and stabilized with titanium trauma splints, wire-composite splints, orthodontic appliances, or sutures, depending on the clinical situation, according to the surgeon’s decision; splinting duration was recorded for each case. Postoperative analgesics and antibiotics were prescribed at the individual surgeon’s discretion. All patients rinsed with 0.2% chlorhexidine for at least one week and followed a soft diet for two weeks. Root canal treatment was not performed prophylactically, but reactively in response to clinical (pain, swelling, suppuration, sinus tract) and/or radiological (apical radiolucency, external root resorption) signs of pulpal infection in the subsequent follow-up appointments.

### Clinical examination

The clinical examination for this study included visual inspection of the transplant site for signs of inflammation, assessment of pain on percussion, evaluation of tooth mobility, and sensitivity on CO_2_-snow. Probing depths and gingival recession were measured using a periodontal probe (in mm), and the presence of bleeding or suppuration on probing was recorded at three buccal and three lingual sites per tooth. Tooth discoloration, occlusal contacts, and clinical signs of ankylosis were also assessed. When present, restorations were evaluated for structural integrity and functional adequacy. Standardized photographs of the region of interest were taken from occlusal and lateral perspectives using the same digital camera system (Canon EOS 7D, Melville, NY, USA), equipped with a 60 mm macro lens (Canon EF-S 60 mm, Melville, NY, USA) and a macro ring flash (MF-R76, GODOX Photo Equipment Co., Shenzhen, China). Camera settings were standardized with an aperture of f/25, ISO 200, and a shutter speed of 1/160 s. Additionally, full-arch intraoral scans of both the maxilla and mandible were obtained using the TRIOS Move+ system (3Shape, Copenhagen, Denmark).

### Digital data evaluation

Periapical radiographs of the transplanted teeth were taken using the parallel technique with a holder and an intraoral sensor (Xios XG Supreme, Dentsply Sirona Schweiz AG, Baden, Switzerland). The radiographs immediately following surgery and at follow-up, clinical images, and relevant transplant information were compiled and uploaded to a dedicated online evaluation platform. Independent assessment of the radiograph at follow-up was performed by a panel of six examiners, comprising three board-certified OS and three GPs, each with at least five years of clinical experience. Prior to assessment, examiners completed a calibration exercise using reference cases to align the scoring criteria. Raters were blinded to one another’s ratings. The following radiographic parameters were evaluated:

1. Presence of radiolucency (periapical, pararadicular, intraradicular, intracoronal).

2. Continuity of the periodontal ligament space.

3. Obliteration of the pulp canal and chamber.

4. Evidence of root development following surgery.

5. Horizontal and vertical bone loss.

Each parameter was scored on a three-point scale: “yes,” “no,” or “unsure”. To assess intra-rater reliability, 10% of cases were randomly interspersed as duplicates within each examiner’s case set.

### Survival and success criteria

**Fig. 1:**
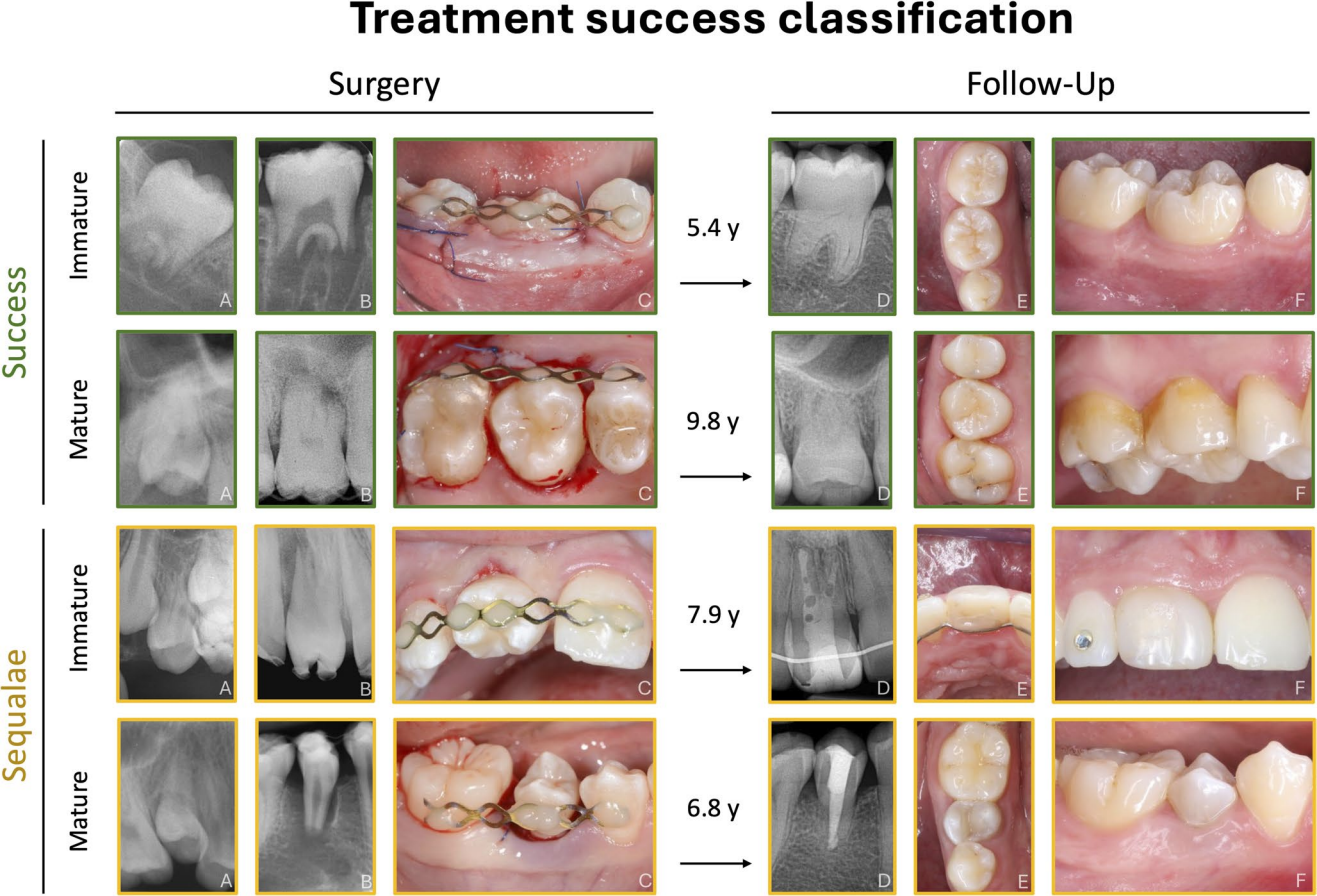
Representative cases illustrating treatment outcomes (successes/green and sequelae/yellow) across study groups at the time of surgery and follow-up. Donor and recipient teeth were as follows: 48 → 46 (immature, success), 18 → 16 (mature, success), 24 → 11 (immature, sequelae: root canal treatment), and 25 → 45 (mature, sequelae: replacement resorption and root canal treatment)

Survival and success of autotransplanted teeth were evaluated using criteria consistent with previous studies (Fig. 1) [20, 26, 27]. Survival was defined as the tooth remaining in place at the follow-up examination. Success was defined based on a predefined outcome set comprising:

1. Clinical criteria: absence of inflammation (e.g., swelling, sinus tract, or pain), no pain on percussion, tooth in occlusion, no need for root canal treatment, probing depths < 3.5 mm, and tooth mobility not exceeding grade I.

2. Radiographic criteria: continuous periodontal ligament space, absence of inflammatory or replacement root resorption, absence of periapical or pararadicular radiolucency, and pulp canal obliteration or bone ingrowth into the pulp chamber in transplants without root canal treatment.

The fulfillment of radiographic criteria for the success analysis was based on the consensus of the OS panel, selected as the reference standard, given their specialist training in this procedure. When no consensus was reached, images were reviewed and classified by an agreement by three of the authors (T.Z., E.C.Q., and C.R.).

Outcomes were assessed against cohort subsets defined by available data. Survival was calculated across the full cohort, including patients examined at follow-up, those reporting via phone, and documented losses in the patient charts. Success was restricted to patients examined at follow-up and documented losses, as clinical and radiological assessment was required. Revascularization rates were derived exclusively from patients examined with radiographic images at follow-up (Table 1).

**Table 1:** Overview of denominators applied per outcome, reflecting differences in data availability across the study cohort. FUP = follow-up examination; PI = phone inquiry

Outcome	Denominator	Immature *N* = 39	Mature *N* = 19
Survival	Full cohort (FUP + phone inquiry + losses)	26 + 9 + 4	11 + 3 + 5
Success	FUP + losses	26 + 4	11 + 5
Revascularization	FUP only	26	11

### Statistical analysis

All analyses in this report were performed with the statistics software R, version 4.4.2 [28]. Patient-related, tooth-related, surgery-related, clinical, and radiographic outcomes at the follow-up were descriptively summarized for the study groups “immature teeth” and “mature teeth” by providing means, medians, and standard deviations for continuous variables and by giving frequencies and percentages for categorical variables.

Since some patients had more than one transplant, patient-related, tooth-related, surgery-related, and clinical outcomes at the follow-up were compared between study groups with the help of linear mixed regression models (continuous outcomes) or GEE logistic regression models (binary outcomes). In the linear mixed regression models, the patient was modeled as a random intercept, and goodness-of-fit of models was assessed and confirmed by the tools of the DHARMa package by Hartig [29]. In the generalized estimating equation (GEE) models, the covariance structure was chosen as “exchangeable,” and goodness-of-fit of models was assessed and confirmed by the Le Cessie-van Houwelingen-Copas-Hosmer test [30].

GEE models, going along the abovementioned procedure, were furthermore used to assess what parameters are significantly associated with transplant survival and transplant success (success vs. sequelae, only teeth observed at follow-up). Only univariable analyses were conducted as the number of failures and successes was small.

The percentage of full agreement in radiographic evaluation, including two-sided 95% Wilson Cis, was calculated for each clinician group and overall. Rates of full agreement between clinician groups were furthermore tested for differences with McNemar’s test. Finally, all p-values lower than or equal to 0.05 were considered statistically significant.

## Results

### Study sample and survival analysis

Fifty patients (35 female, 15 male; mean age 22.9 ± 5.5 years) with 58 transplanted teeth were included in the survival analyses; of these, 12 patients (with 12 transplanted teeth) confirmed transplant survival by telephone but declined clinical follow-up. Thirty-nine immature transplants, with a mean follow-up of 8.4 ± 5.7 years, exhibited root formation stages of ¼ (*n* = 9), ½ (*n* = 18), and ¾ (*n* = 12) at the time of surgery and were performed using conventional surgical techniques. Nineteen mature transplants, with a shorter mean follow-up of 5.6 ± 2.8 years (*p* = 0.01), had completed root and apex formation in all cases (*n* = 19, *p* < 0.001), and underwent intraoperative EORER.

Donor sites were more commonly maxillary (*n* = 34) than mandibular (*n* = 24), comprising primarily premolars (*n* = 26) and third molars (*n* = 21), followed by canines (*n* = 7) and first or second molars (*n* = 4). Recipient sites were predominantly mandibular (*n* = 44) over maxillary (*n* = 14), most frequently in the premolar region (*n* = 40), followed by molar (*n* = 11), canine (*n* = 6), and incisor (*n* = 1) regions. Both donor and recipient sites differed significantly between study groups, with more donor canines involved in the mature group (*p* = 0.01). However, no statistically significant differences between study groups were observed regarding surgery-related variables, including indication for the procedure, preoperative stage of eruption, use of enamel-matrix-derivatives during surgery, duration of surgery and splinting, type of splint, and type of recipient site (all p$\:\ge\:$0.12, Table 2). Individual patient-, site-, and surgery-related data stratified by study group are summarized in Supplementary Table 1.

**Table 2:** Patient, surgical, and outcome characteristics stratified by study group. Survival data include all patients with available information from phone inquiry (PI) or follow-up examination (FUP); success and clinical outcomes data are restricted to FUP patients only. Continuous data are presented as mean [median] (SD); count data as n (%). *p < 0.05

Characteristic	Data	*N*	Immature teeth *N* = 39	Mature teeth *N* = 19	*p*-value
Gender	FUP + PI	58			0.34
Female			26 (66.7%)	15 (78.9%)	
Male			13 (33.3%)	4 (21.1%)	
Age at surgery [y]	FUP + PI	58	14.8 [14.4] (2.9)	16.5 [14.8] (5.3)	0.21
Age at follow-up [y]	FUP + PI	58	23.2 [22.4] (4.9)	22.2 [18.3] (6.6)	0.34
Time to follow-up [y]	FUP + PI	58	8.4 [6.5] (5.7)	5.6 [6.2] (2.8)	0.01*
Position donor tooth	FUP + PI	58			0.01*
Incisors/Canines			1 (2.6%)	6 (31.6%)	
Premolars/Molars			38 (97.4%)	13 (68.4%)	
Position recipient tooth	FUP + PI	58			0.01*
Incisors/Canines			1 (2.6%)	6 (31.6%)	
Premolars/Molars			38 (97.4%)	13 (68.4%)	
Root development at surgery	FUP + PI	58			< 0.001**
Complete apex formation (Ac)			0 (0.0%)	19 (100.0%)	
R1/4-R3/4			39 (100.0%)	0 (0.0%)	
Preoperative stage of eruption	FUP + PI	58			0.28
Included/Dystopic			28 (71.8%)	12 (63.2%)	
Partially erupted/Erupted			11 (28.2%)	7 (36.8%)	
Indication	FUP + PI	58			0.23
Aplasia, caries and other			30 (76.9%)	11 (57.9%)	
Dystopia/Malposition			9 (23.1%)	8 (42.1%)	
Enamel-Matrix-Derivate	FUP + PI	58			0.20
No			29 (74.4%)	11 (57.9%)	
Yes			10 (25.6%)	8 (42.1%)	
Duration of surgery [min]	FUP + PI	55	102.4 [90.0] (44.7)	104.7 [90.0] (38.9)	0.91
Duration of splinting [days]	FUP + PI	53	43.4 [35.5] (27.7)	65.1 [39.0] (53.4)	0.17
Splint types	FUP + PI	58			0.86
Orthodontic. appliance/Wire composite splint			9 (23.1%)	4 (21.1%)	
Suture/Titanium trauma splint			30 (76.9%)	15 (78.9%)	
Type of recipient site	FUP + PI	56			0.12
Extraction socket			2 (5.4%)	4 (21.1%)	
Pristine ridge			35 (94.6%)	15 (78.9%)	
Survival	FUP + PI	58			0.18
No			4 (10.3%)	5 (26.3%)	
Yes			35 (89.7%)	14 (73.7%)	
Pain	FUP + PI	49			0.11
No			35 (100.0%)	13 (92.9%)	
Yes			0 (0.0%)	1 (7.1%)	
Success	FUP	37			0.11
No (= Sequelae)			7 (26.9%)	6 (54.5%)	
Yes			19 (73.1%)	5 (45.5%)	
Presence of restorations	FUP	37			0.99
No			19 (73.1%)	8 (72.7%)	
Yes			7 (26.9%)	3 (27.3%)	
Root canal treatment	FUP	37			0.24
No			23 (88.5%)	8 (72.7%)	
Yes			3 (11.5%)	3 (27.3%)	
Infection signs	FUP	37			0.11
No			26 (100.0%)	10 (90.9%)	
Yes			0 (0.0%)	1 (9.1%)	
Tooth mobility	FUP	37			0.19
No			25 (96.2%)	9 (81.8%)	
Yes			1 (3.8%)	2 (18.2%)	
Sensitivity on CO_2_-snow	FUP	37			0.38
No			23 (88.5%)	9 (81.8%)	
Yes			3 (11.5%)	2 (18.2%)	
Infraposition	FUP	37			0.90
No			23 (88.5%)	10 (90.9%)	
Yes			3 (11.5%)	1 (9.1%)	
Maximum probing depth [mm]	FUP	37	2.9 [3.0] (0.6)	3.5 [3.0] (1.5)	0.25
Gingival recession	FUP	37			0.02*
No			22 (84.6%)	5 (45.5%)	
Yes			4 (15.4%)	6 (54.5%)	
Bleeding on probing	FUP	37			0.81
No			13 (50.0%)	5 (45.5%)	
Yes			13 (50.0%)	6 (54.5%)	

Survival rates were 89.7% for immature transplants, with 4 losses recorded between 4.1 and 8.3 years after the intervention, and 73.7% for mature transplants, with 5 losses recorded 1.5 to 6.9 years post-surgery. Causes of transplant failure included replacement resorption (*n* = 1 immature, *n* = 3 mature), inflammatory root resorption (*n* = 1 immature, *n* = 1 mature), invasive cervical resorption (*n* = 1 immature, *n* = 1 mature), and apicomarginal lesion (*n* = 1 immature) (Table 3). Univariate analysis identified longer splinting duration (OR 1.03; CI 1.00, 1.05, *p* = 0.046) and pristine alveolar ridge compared to fresh extraction sites (OR 16.6; CI 2.20, 125; *p* = 0.006) as significant predictors of increased transplant survival (Table 4).

**Table 3:** Summary of periodontal and endodontic sequelae and losses observed in immature and mature transplants

Category	Immature (Follow-up + Losses) *N* = 30		Mature (Follow-up + Losses) *N* = 16	
	Periodontal	Endodontic	Periodontal	Endodontic
Sequelae at follow-up	4 (13.3%)	3 (10.0%)	1 (6.3%)	5 (31.3%)
Root canal treatment	—	3 (10.0%)	—	3 (18.8%)
Infraposition/Ankylosis	2 (6.7%)	—	—	—
Replacement resorption	1 (3.3%)	—	1 (6.3%)	—
Invasive cervical resorption	1 (3.3%)	—	—	—
Apical periodontitis	—	—	—	1 (6.3%)
Apicomarginal lesion	—	—	—	1 (6.3%)
Losses before follow-up	3 (10.0%)	1 (3.3%)	5 (31.3%)	0 (0.0%)
Replacement resorption	1 (3.3%)	—	4 (25.0%)	—
Invasive cervical resorption	1 (3.3%)	—	1 (6.3%)	—
Inflammatory root resorption	1 (3.3%)	—	—	—
Apicomarginal lesion	—	1 (3.3%)	—	—

**Table 4:** Univariable screening of survival and success factors available *p < 0.05

Characteristic	Odds Ratio Survival	*p*-value	Odds Ratio Success	*p*-value
Group		0.18		0.11
Immature	Baseline		Baseline	
Mature	0.33 (0.07, 1.69)		0.29 (0.06, 1.29)	
Gender		0.22		0.69
Female	Baseline		Baseline	
Male	0.40 (0.09, 1.71)		1.44 (0.24, 8.85)	
Age at Surgery [y]	0.99 (0.82, 1.19)	0.89	0.98 (0.84, 1.13)	0.76
Position donor tooth		0.76		0.11
Incisors/Canines	Baseline		Baseline	
Premolars/Molars	1.44 (0.15, 14.0)		7.13 (0.65, 78.0)	
Position recipient tooth		0.76		0.047*
Incisors/Canines	Baseline		Baseline	
Premolars/Molars	1.44 (0.15, 14.0)		10.7 (1.04, 111)	
Preoperative stage of eruption		0.13		0.40
Included/Dystopic	Baseline		Baseline	
Partially erupted/Erupted	0.21 (0.03, 1.61)		0.53 (0.13, 2.28)	
Indication		0.19		0.62
Aplasia, caries and other	Baseline		Baseline	
Dystopia/Malposition	0.37 (0.08, 1.64)		0.65 (0.12, 3.57)	
Enamel-Matrix-Derivate		0.19		0.52
No	Baseline		Baseline	
Yes	4.31 (0.49, 38.2)		1.62 (0.38, 6.98)	
Duration of surgery [min]	1.00 (0.98, 1.02)	0.91	1.02 (0.99, 1.04)	0.12
Duration of splinting [days]	1.03 (1.00, 1.05)	0.046*	0.99 (0.97, 1.01)	0.09
Splint types		0.90		0.62
Orthodontic appl./Wire comp. splint	Baseline		Baseline	
Suture/Titanium trauma splint	0.89 (0.16, 5.09)		0.67 (0.14, 3.27)	
Type of recipient site		0.006**		n/a
Extraction socket	Baseline		n/a	
Pristine ridge	16.6 (2.20, 125)		n/a	

### Clinical findings

Among the included patients, 33 (26 female and 7 male) with a mean age of 24.3 ± 5.9 years and 37 transplanted teeth in function consented to a clinical and radiographic follow-up examination (Fig. 1). These comprised 26 immature transplants with a mean follow-up of 8.3 ± 6.0 years and 11 mature transplants with a mean follow-up of 6.8 ± 2.7 years. At follow-up, the vast majority of transplants were asymptomatic and showed no signs of infection (*n* = 36, 97.3%). One root canal-treated transplant (mature group) was associated with pain, and one canine (mature group) presented with a sinus tract (each *n* = 1, 2.7%). These two teeth, together with one tooth from the immature group, had probing depths exceeding 5 mm; all remaining teeth showed periodontal probing depths within the physiologic range. Mean BOP was 15.3 ± 18.6%, and tooth mobility was grade 0 or 1 across all teeth. Cold sensitivity was recorded in five teeth (*n* = 3 immature, *n* = 2 mature). Six teeth had undergone postoperative root canal treatment (*n* = 3 immature, *n* = 3 mature), and restorations were present in 10 teeth (*n* = 7 immature, *n* = 3 mature). Four teeth, all in the mandibular premolar region, were in infraposition (*n* = 3 immature, *n* = 1 mature). Gingival recessions were more frequently observed in mature than immature transplants (*n* = 6 vs. *n* = 4; 54.5% vs. 15.4%; *p* = 0.02). All other clinical outcomes did not differ significantly between the two groups (*p* ≥ 0.11).

### Radiographic findings, interrater agreement, and intrarater repeatability

**Fig. 2:**
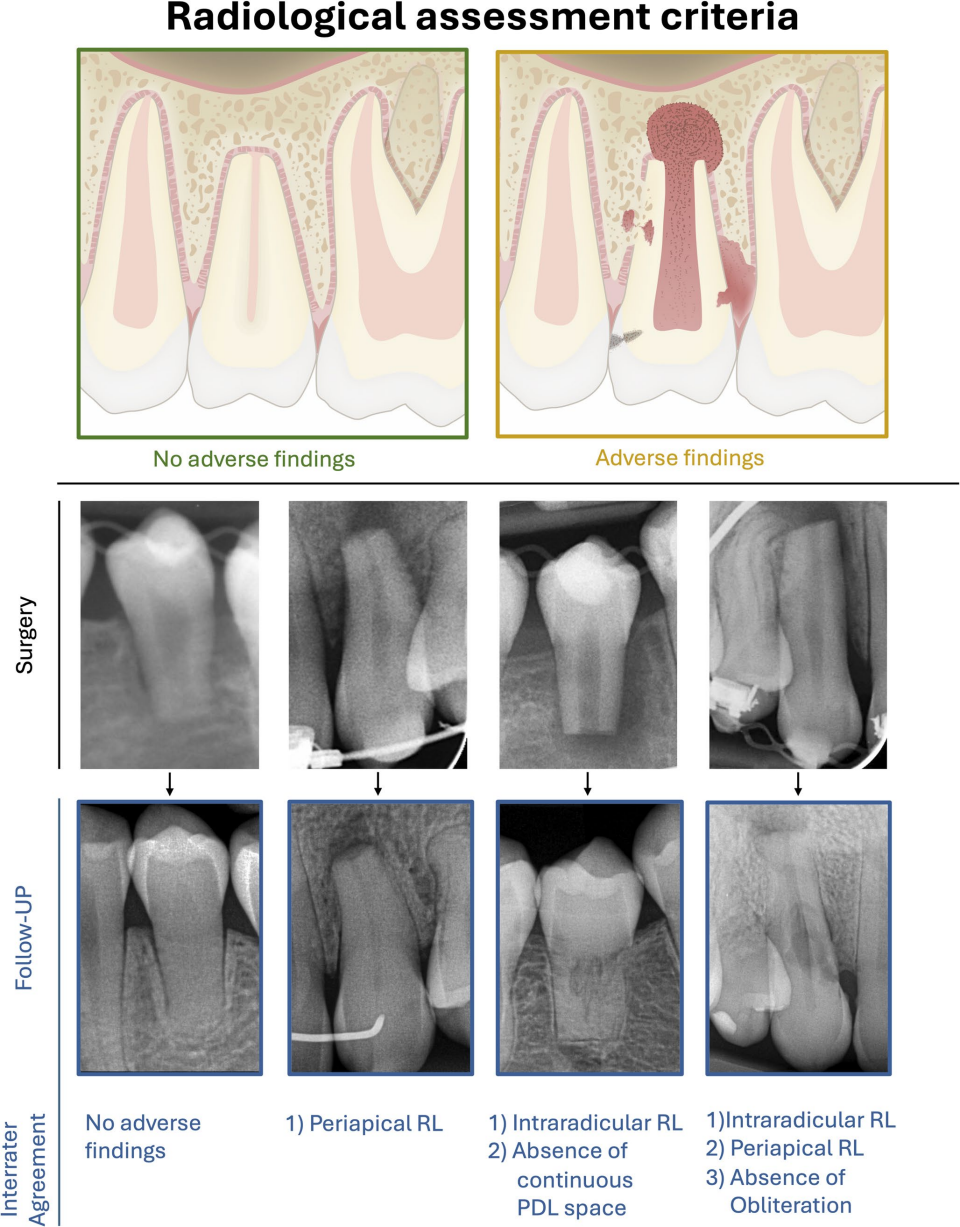
Radiological assessment criteria applied by the six examiners. The schematics illustrate a tooth without adverse findings (green) and one with adverse findings (orange), based on periodontal ligament (PDL) space continuity, pulp canal obliteration, radiolucency (RL; intracoronal, intraradicular, pararadicular, periapical), and marginal bone loss. Paired radiographs of four representative cases from the mature group, obtained at surgery and at follow-up. Along with the adverse findings identified by interrater agreement

Radiographic findings across 222 ratings (37 teeth, six examiners) are summarized in Table 5. A continuous periodontal ligament space was the most frequent finding (79.3%), with identical rates between OS and GPs. Pulp canal obliteration was identified more often by OS than by GPs (70.3% vs. 45.9%), whereas progressive root development was rated comparably by both groups (35.1% vs. 34.2%). Pathological findings were uncommon overall; when identified, they were generally reported more often by GPs than by OS, most notably periapical radiolucency (12.6% vs. 4.5%), vertical bone loss (14.4% vs. 2.7%), and horizontal bone loss (8.1% vs. 2.7%). GPs also flagged an indication for further treatment three times as often as OS (5.4% vs. 1.8%). Full inter-rater agreement was lowest for pulp canal obliteration (29.7%), the only item with a statistically significant between-group difference, with OS showing approximately 30% points higher agreement than GPs (*p* = 0.02). The highest inter-rater agreement was observed for pararadicular and intracoronal radiolucency (both 83.8%, Table 6; Fig. 2).

**Table 5:** Radiographic ratings of 37 transplanted teeth at follow-up by six examiners, stratified by clinician background (three general practitioners [GP] and three oral surgeons [OS])

Item	*N* (ratings)	Overall	GP	OS
Periapical radiolucency	222			
No		192 (86.5%)	89 (80.2%)	103 (92.8%)
Unsure		11 (5.0%)	8 (7.2%)	3 (2.7%)
Yes		19 (8.6%)	14 (12.6%)	5 (4.5%)
Pararadicular radiolucency	222			
No		211 (95.0%)	101 (91.0%)	110 (99.1%)
Unsure		4 (1.8%)	4 (3.6%)	0 (0.0%)
Yes		7 (3.2%)	6 (5.4%)	1 (0.9%)
Intraradicular radiolucency	222			
No		208 (93.7%)	106 (95.5%)	102 (91.9%)
Unsure		2 (0.9%)	0 (0.0%)	2 (1.8%)
Yes		12 (5.4%)	5 (4.5%)	7 (6.3%)
Intracoronal radiolucency	222			
No		204 (91.9%)	102 (91.9%)	102 (91.9%)
Unsure		3 (1.4%)	1 (0.9%)	2 (1.8%)
Yes		15 (6.8%)	8 (7.2%)	7 (6.3%)
Continuous periodontal ligament space	222			
No		21 (9.5%)	13 (11.7%)	8 (7.2%)
Unsure		25 (11.3%)	10 (9.0%)	15 (13.5%)
Yes		176 (79.3%)	88 (79.3%)	88 (79.3%)
Continuous root development	222			
No		130 (58.6%)	68 (61.3%)	62 (55.9%)
Unsure		15 (6.8%)	5 (4.5%)	10 (9.0%)
Yes		77 (34.7%)	38 (34.2%)	39 (35.1%)
Pulp obliteration	222			
No		78 (35.1%)	55 (49.5%)	23 (20.7%)
Unsure		15 (6.8%)	5 (4.5%)	10 (9.0%)
Yes		129 (58.1%)	51 (45.9%)	78 (70.3%)
Horizontal bone loss	222			
No		207 (93.2%)	100 (90.1%)	107 (96.4%)
Unsure		3 (1.4%)	2 (1.8%)	1 (0.9%)
Yes		12 (5.4%)	9 (8.1%)	3 (2.7%)
Vertical bone loss	222			
No		200 (90.1%)	93 (83.8%)	107 (96.4%)
Unsure		3 (1.4%)	2 (1.8%)	1 (0.9%)
Yes		19 (8.6%)	16 (14.4%)	3 (2.7%)
Intention to treat	222			
No		204 (91.9%)	99 (89.2%)	105 (94.6%)
Unsure		10 (4.5%)	6 (5.4%)	4 (3.6%)
Yes		8 (3.6%)	6 (5.4%)	2 (1.8%)

**Table 6:** Percentages of full agreement with 95%-CI at follow-up by six examiners, stratified by clinician background (three general practitioners [GP] and three oral surgeons [OS]). *p < 0.05

Item	Overall	GP	OS	*p*-value
Periapical radiolucency	62.2 (46.1; 75.9)	67.6 (51.5; 80.4)	86.5 (72.0; 94.1)	0.07
Pararadicular radiolucency	83.8 (68.9; 92.3)	83.8 (68.9; 92.3)	97.3 (86.2; 99.5)	0.07
Intraradicular radiolucency	81.1 (65.8; 90.5)	94.6 (82.3; 98.5)	81.1 (65.8; 90.5)	0.07
Intracoronal radiolucency	83.8 (68.9; 92.3)	89.2 (75.3; 95.7)	86.5 (72.0; 94.1)	1.00
Continuous periodontal ligament space	32.4 (19.6; 48.5)	51.4 (35.9; 66.6)	51.4 (35.9; 66.6)	1.00
Continued root development	54.1 (38.4; 69.0)	73.0 (57.0; 84.6)	64.9 (48.8; 78.2)	0.55
Pulp obliteration	29.7 (17.5; 45.8)	48.6 (33.4; 64.1)	78.4 (62.8; 88.6)	0.02*
Horizontal bone loss	73.0 (57.0; 84.6)	75.7 (59.9; 86.6)	91.9 (78.7; 97.2)	0.07
Vertical bone loss	70.3 (54.2; 82.5)	75.7 (59.9; 86.6)	91.9 (78.7; 97.2)	0.07
Intention to treat	70.3 (54.2; 82.5)	75.7 (59.9; 86.6)	86.5 (72.0; 94.1)	0.29

Intra-rater repeatability was generally higher among OS (range 62.5%−100%) than among GPs (50.0%−100%). Continuous periodontal ligament space was the least reproducible item in both groups (62.5% in OS, 50% in GPs), while pararadicular, intraradicular, and intracoronal radiolucency were reproduced at 100% by all six examiners.

### Success analysis and associations

Of the 37 teeth assessed at the follow-up examination, 13 did not meet the predefined success criteria (*n* = 7 immature, *n* = 6 mature). When transplant losses are additionally taken into account, this corresponds to overall success rates of 63.3% for immature and 31.3% for mature transplants. Observed sequelae included replacement resorption (*n* = 1 immature, *n* = 1 mature), invasive cervical resorption (*n* = 1 immature), infraposition (*n* = 2 immature), root canal treatment (*n* = 3 immature, *n* = 3 mature), apical periodontitis (*n* = 1 mature), and apicomarginal lesion (*n* = 1 mature). Of note, seven of these 13 teeth presented with two or more concurrent adverse findings. Clinical-radiographic findings compatible with pulp revascularization (no signs of infection, no need for RCT, no periapical radiolucency, presence of pulp canal obliteration) were observed in 23 immature and 6 mature transplants (88.5% vs. 54.5%). On univariate analysis, premolar and molar recipient sites were identified as a significant predictor of increased transplant success (OR 10.7; CI 1.04, 111; *p* = 0.047; Table 4).

## Discussion

This retrospective cohort study evaluated the long-term outcomes of conventional immature tooth autotransplantation and mature tooth autotransplantation involving root-end resection, based on clinical and radiographic follow-up findings supplemented by survival assessment of non-attendees. Immature transplants demonstrated higher survival (89.7% vs. 73.7%) and success rates (63.3% vs. 31.3%) than mature transplants, with revascularization rates of 88.5% and 54.5%, respectively. Longer splinting duration and pristine ridge recipient sites were associated with an increased transplant survival, while premolar and molar recipient sites were associated with higher treatment success. Radiographic pathological findings were infrequent overall, though inter-rater agreement varied considerably across assessed items, with OS consistently yielding consistently higher agreement and repetability than GPs.

Although tooth replacement via dental implants has demonstrated excellent long-term success across a wide range of clinical scenarios, their use in younger patients remains controversial due to ongoing craniofacial growth of jaw bones especially in the anterior maxilla [31–35]. In this context, autotransplantation is of particular clinical relevance in growing patients, for whom implant therapy is not yet indicated. By preserving a functional periodontal ligament, autotransplantation supports continued development of the surrounding hard- and soft-tissue while maintaining the potential for alveolar growth and orthodontic toot movement [2, 6].

The survival rates observed for immature teeth in the present study are slightly lower than those reported in recent reviews, which describe 5- to 10-year survival rates exceeding 95% [36–38]. This discrepancy may be partly explained by the high proportion of molar donor teeth in our cohort, as survival rates vary considerably by tooth type. Premolar transplants have been reported to achieve the most favorable survival rates (95.4%), followed by molars (86.6%), and anterior teeth (75.8%), potentially reflecting simpler root anatomy and less demanding surgical handling of premolars [20, 22]. In the present cohort, losses amongst immature transplants occurred due to periodontal complications in three cases (2 premolars, 1 molar) and endodontic complications in one molar.

Similarly, the lower survival rate observed for mature transplants is consistent with the existing literature, which reports less favorable outcomes in teeth with complete mature root formation [17, 22, 39]. All five failures in the mature group were attributable to periodontal complications (2 molars, 2 canines, 1 premolar). A systematic review of mature transplants, in which perioperative root canal treatment was performed in the majority of cases, reported a higher 5-year survival rate than observed in the present cohort (90.5% vs.73.7%) [17]. Part of this discrepancy may stem from the telephone-based survival assessment, allowing detection of failures among patients who did not return for clinical follow-up, a well-recognized source of attrition bias in dental studies [40, 41].

The success rates observed in this study are also lower than those reported in the literature, ranging from 84% to 96.6% [18, 36, 39, 42]. While survival differences between groups are modest in absolute terms, mature teeth are considerably more susceptible to postoperative complications, which is reflected in the lower success rates [18, 39, 43]. Endodontic complications arise when pulp revascularization fails and were observed in four immature and five mature teeth, including one loss among immature teeth. Pulpal healing is closely related to apical foramen dimensions, with immature teeth showing markedly higher healing rates [15]. In mature teeth, the apical constriction limits the surface area available for revascularization, providing the rationale for extraoral root-end resection, an emerging technique aimed at promoting pulp revascularization and potentially obviating root canal treatment [19]. Preclinical models and histological studies indicate that apicoectomy can facilitate revascularization in mature teeth, with fibrovascular connective tissue replacing the pulp, whereas non-apicoectomized mature teeth may fail to revascularize [44–46]. At follow-up of the present cohort, 23 of 26 immature and 6 of 11 mature transplants assessed at follow-up showed radiographic signs of revascularization (88.5% and 54.5%, respectively). These rates were marginally lower than the revascularization rates exceeding 90% reported for immature donor teeth [15], yet fall within the 44.4–100% range documented for the limited clinical evidence available on EORER [20, 21, 47], and substantially exceed the 16% rate observed when the apical constriction is left intact [15]. Positive cold sensitivity in three immature and two mature transplants at follow-up provides adjunctive clinical support for potential functional pulpal healing. Nervertheless, it is important to acknowledge, that significant heterogeneity exists in how success is defined across dental autotransplantation studies, creating challenges cross-study comparisons [27, 38, 48].

Periodontal complications are fundamentally driven by damage to the root cementum during extraction, requiring greater forces in mature teeth with fully developed periodontal ligaments, and damage to root tissues during extraoral handling and storage [5, 16, 49, 50]. In the present study, periodontal sequelae, including replacement resorption, invasive cervical resorption, and infraposition indicative of ankylosis, were observed in seven immature and seven mature teeth, including 8 out of 9 losses attributable to periodontal complications. Strategies to mitigate these risks include minimally traumatic extraction techniques, minimizing extraoral dry time through computer-assisted workflows and rapid prototyping, and intermediate storage in cell culture media [49–54]. Infraposition was observed in four transplants (3 immature, 1 mature), all in the mandibular premolar region, and might be ankylosis-related. This becomes particularly relevant in growing patients who have not yet completed vertical alveolar development at the time of surgery, whereas mature transplants are placed into a jaw that has largely ceased vertical growth [55, 56]. These findings highlight the importance of carefully timing transplantation surgery in relation to craniofacial growth and underscore the need for long-term monitoring for progressive infraposition that may require further intervention.

In the present cohort, longer splinting duration and pristine ridge recipient sites were associated with increased survival, while premolar and molar recipient sites were the only significant predictors for higher success rates. These findings should be interpreted cautiously: the number of adverse events was limited, analyses were restricted to univariable models, confidence intervals were wide. In addition, the more complex mature-tooth transplants were overrepresented at anterior recipient sites, potentially confounding the observed associations. Inferior outcomes at anterior recipient sites were also observed in another study [50]. However, another investigation reported on excellent anterior outcomes in a pediatric cohort involving predominantly single-rooted premolar donors in fresh sockets with adequate bone [57]. Several established prognostic factors, including patient age, extraoral time, donor and recipient position, eruption stage, root formation stage, donor-tooth type, and antibiotic prophylaxis did not reach significance in our analysis. This likely reflects the limited sample size and statistical power rather than a true absence of biological or clinical effect [23, 39, 48–50].

A distinctive feature of this study is the comparison of periapical radiograph assessment between GPs and OS, which, to the best of the authors’ knowledge, has not been previously investigated in the context of tooth autotransplantation. This is clinically relevant, as many autotransplants are followed up in general dental practice, where variability in recognizing healing complications may affect timely intervention. Agreement was highest for pararadicular and intracoronal radiolucency (83.8%) and lowest for pulp obliteration (29.7%), the only item with a statistically significant difference between groups (*p* = 0.02), with OS achieving approximately 30% points higher agreement. As obliteration is generally regarded as a favorable healing sign, its misinterpretation could lead to unnecessary endodontic intervention. Across several other items, including periapical, pararadicular, and intraradicular radiolucency, as well as horizontal and vertical bone loss, GPs consistently reported pathological findings and intention to treat more frequently than OS, with differences approaching but not reaching statistical significance (all *p* ≥ 0.07), possibly indicating a lower threshold for identifying abnormalities or a higher rate of false-positive interpretations. Intrarater repeatability was overall higher among OS, whereas the continuous periodontal ligament space was the least reproducible finding for both groups, possibly reflecting the difficulty of assessing subtle periodontal ligament changes on two-dimensional radiographs. These findings highlight the potential for diagnostic variability when follow-up is conducted outside specialist settings and support the use of standardized assessment protocols, calibration exercises, and OS consultation before any radiographic finding is translated into a treatment decision [58].

Several limitations of the present investigation should be acknowledged. The retrospective design limits control over confounding variables, while the relatively small sample size restricts analyses to univariate models. The unequal distribution of immature and mature transplants, combined with a significantly shorter follow-up for the mature group (5.6 vs. 8.4 years; *p* = 0.01), may limit direct comparability and underestimate late complications. All surgeries were performed at a single academic center over 22 years, during which protocols may have evolved, potentially introducing temporal confounding. Telephone-based survival assessment, while effective in reducing attrition bias, lacks clinical verification and may be subject to recall bias. Radiographic assessment was limited to two-dimensional periapical radiographs, which have inherent limitations in detecting early changes compared to 3-dimensional imaging modalities such as cone-beam computed tomography, and the inter-rater agreement analysis included only three examiners per group, limiting generalizability. Finally, the composite success criteria and the classification of certain findings remain subject to debate, which may affect comparability with studies using different outcome definitions. Hence, future research should focus on prospective multicenter studies with larger samples and longer follow-up to enable multivariable analyses, apply standardized outcome definitions to improve cross-study comparability, and include broader inter-rater agreement studies to inform calibration tools and follow-up guidelines for general dental practice.

## Conclusions

Within the limitations of this study, the following conclusions can be drawn:

- Autotransplantation of immature transplants demonstrated higher survival and success rates than mature transplants treated with EORER.
- Periodontal complications account for most transplant failures in both groups.
- Despite lower overall outcomes, pulp revascularization was observed in more than half of mature EORER-treated transplants, supporting the biological potential of this approach and its possible role in reducing the need for prophylactic root canal treatment.
- Radiographic pathological findings were overall infrequent; however, considerable variability existed in radiographic interpretation between oral surgeons and general practitioners.
- Oral surgeons demonstrated higher inter- and intrarater agreement than general practitioners, highlighting the need for standardized radiographic evaluation criteria and calibration in long-term follow-up.

## Supplementary Information

Below is the link to the electronic supplementary material.

Supplementary Material 1 (DOCX 53.4 KB)

## Data Availability

The data that support the findings of this study are available from the corresponding author upon reasonable request.

## Declarations

### Generative AI and AI-assisted technologies in the writing process

During the preparation of this work, the authors used ChatGPT/OpenAI for language editing purposes. After using this tool, the authors reviewed and edited the content as needed and took full responsibility for the content of the publication.
