# Supplementary material for: Long-term survival and success rates of immature versus root-end resected mature tooth autotransplants: part I of a retrospective cohort study
Source: Clin Oral Investig. 2026 Jul 7;30(8):310. doi: 10.1007/s00784-026-06990-w (PMC13342390; doi:10.1007/s00784-026-06990-w)
Supplement: Supplementary file 1 — Supplementary Material 1 (DOCX 53.4 KB) [file 784_2026_6990_MOESM1_ESM.docx]

**Supplementary Table**

| **Patient ID** | **Study ID** | **Data** | **Study group** | **Survival** | **Success / Sequalae** | **Gender** | **Age at  surgery** | **Age at  follow-up** | **Follow-Up  Interval** | **Stage of Root  Development preoperative** | **Stage of Eruption  preoperative** | **Donor  site** | **Recipient  site** | **Type of  Recipient Site** | **Use of  EMD** | **Duration of  Surgery (min)** | **Type of  Splint** | **Duration of  Splinting (d)** | **Pain** | **Signs of infection** | **Max. Gingival Recession (mm)** | **Max.  Probing  Depth (mm)** | **BOP %** | **Sensitivity  on Cold** | **Tooth Mobility** | **Infra- position** | **Presence of Restoration** |
| --- | --- | --- | --- | --- | --- | --- | --- | --- | --- | --- | --- | --- | --- | --- | --- | --- | --- | --- | --- | --- | --- | --- | --- | --- | --- | --- | --- |
| 2 | 2 | FUP | Immature | yes | Success | Female | 20.7 | 22.2 | 1.4 | R2/4 | Included | 28 | 35 | Pristine ridge | no | 150 | TTS | 48 | no | no | 1 | 3 | 0.0 | yes | 0 | no | no |
| 3 | 3 | FUP | Immature | yes | Success | Female | 17.4 | 19.1 | 1.6 | R1/4 | Included | 18 | 15 | Pristine ridge | no | 150 | TTS | 45 | no | no | 0 | 3 | 50.0 | yes | 0 | no | no |
| 3 | 4 | FUP | Immature | yes | Success | Female | 17.4 | 19.1 | 1.6 | R2/4 | Included | 28 | 25 | Pristine ridge | no | 150 | TTS | 45 | no | no | 0 | 3 | 16.7 | yes | 0 | no | no |
| 5 | 6 | FUP | Immature | yes | Success | Male | 12.3 | 31.8 | 19.4 | R1/4 | Included | 14 | 24 | Pristine ridge | no | 50 | WCS | 30 | no | no | 0 | 3 | 50.0 | no | 0 | no | Composite |
| 10 | 11 | FUP | Immature | yes | Success | Male | 19.2 | 21.3 | 2.1 | R1/4 | Included | 28 | 35 | Pristine ridge | yes | 120 | TTS | 62 | no | no | 0 | 2 | 16.7 | no | 0 | no | no |
| 12 | 13 | FUP | Immature | yes | Success | Male | 18.3 | 32.5 | 14.3 | R3/4 | Included | 28 | 15 | Pristine ridge | no | 70 | OA | 42 | no | no | 0 | 2 | 33.3 | no | 0 | no | Composite |
| 13 | 14 | FUP | Immature | yes | Success | Female | 16.9 | 22.0 | 5.0 | R2/4 | Included | 38 | 35 | Pristine ridge | no | 135 | OA | 28 | no | no | 2 | 3 | 0.0 | no | 0 | no | no |
| 13 | 15 | FUP | Immature | yes | Success | Female | 16.9 | 22.0 | 5.0 | R1/4 | Included | 48 | 45 | Pristine ridge | no | 135 | OA | 28 | no | no | 1 | 2 | 0.0 | no | 0 | no | no |
| 16 | 18 | FUP | Immature | yes | Success | Female | 18.4 | 23.8 | 5.3 | R2/4 | Included | 18 | 36 | Pristine ridge | no | 120 | TTS | 29 | no | no | 0 | 3 | 0.0 | no | 0 | no | no |
| 17 | 19 | FUP | Immature | yes | Success | Female | 12.0 | 15.9 | 3.9 | R1/4 | Included | 15 | 45 | Pristine ridge | yes | 75 | TTS | 75 | no | no | 0 | 3 | 0.0 | no | 0 | no | no |
| 20 | 23 | FUP | Immature | yes | Success | Female | 12.0 | 22.4 | 10.3 | R3/4 | Erupted | 35 | 25 | Pristine ridge | no | 90 | TTS | 25 | no | no | 0 | 3 | 0.0 | no | 0 | no | no |
| 21 | 24 | FUP | Immature | yes | Success | Female | 16.5 | 21.2 | 4.6 | R3/4 | Included | 17 | 45 | Pristine ridge | yes | 70 | TTS | 20 | no | no | 0 | 1 | 0.0 | no | 1 | no | no |
| 22 | 25 | FUP | Immature | yes | Success | Female | 9.8 | 30.6 | 20.8 | R2/4 | Erupted | 14 | 45 | n/a | no | n/a | OA | n/a | no | no | 0 | 3 | 0.0 | no | 0 | no | Partial Crown |
| 23 | 26 | FUP | Immature | yes | Success | Female | 13.3 | 29.8 | 16.3 | R2/4 | Included | 45 | 15 | Pristine ridge | no | 120 | WCS | 33 | no | no | 0 | 3 | 0.0 | no | 0 | no | no |
| 24 | 27 | FUP | Immature | yes | Success | Female | 10.9 | 16.9 | 6.0 | R2/4 | Partially erupted | 15 | 45 | Pristine ridge | yes | 90 | Suture | 21 | no | no | 0 | 3 | 0.0 | no | 0 | no | no |
| 28 | 32 | FUP | Immature | yes | Success | Male | 17.8 | 23.2 | 5.4 | R2/4 | Included | 48 | 46 | n/a | yes | 90 | TTS | n/a | no | no | 0 | 3 | 33.3 | no | 0 | no | no |
| 30 | 34 | FUP | Immature | yes | Success | Male | 14.0 | 20.8 | 6.8 | R3/4 | Dystopic | 45 | 45 | Pristine ridge | yes | 100 | TTS | 58 | no | no | 0 | 3 | 50.0 | no | 0 | no | no |
| 31 | 35 | FUP | Immature | yes | Success | Female | 12.3 | 34.3 | 22.0 | R3/4 | Partially erupted | 44 | 24 | Pristine ridge | no | n/a | WCS | n/a | no | no | 0 | 2 | 16.7 | no | 0 | no | Composite |
| 32 | 36 | FUP | Immature | yes | Success | Female | 10.6 | 28.0 | 17.4 | R3/4 | Partially erupted | 24 | 34 | Pristine ridge | no | 40 | TTS | 35 | no | no | 0 | 3 | 33.3 | no | 0 | no | no |
| 18 | 20 | FUP | Immature | yes | Root canal treatment | Female | 18.8 | 24.9 | 6.2 | R2/4 | Included | 28 | 45 | Pristine ridge | no | 75 | TTS | 28 | no | no | 1 | 3 | 16.7 | no | 0 | no | Composite |
| 20 | 22 | FUP | Immature | yes | Root canal treatment | Female | 12.0 | 22.4 | 10.3 | R3/4 | Erupted | 45 | 15 | Pristine ridge | no | 90 | TTS | 25 | no | no | 0 | 3 | 0.0 | no | 0 | no | Composite |
| 26 | 30 | FUP | Immature | yes | Root canal treatment | Female | 11.2 | 19.1 | 7.9 | R3/4 | Partially erupted | 24 | 11 | Pristine ridge | yes | 60 | TTS | 14 | no | no | 0 | 3 | 0.0 | no | 0 | no | Composite |
| 6 | 7 | FUP | Immature | yes | Replacement root resorption | Male | 12.5 | 14.0 | 1.5 | R3/4 | Included | 15 | 45 | Pristine ridge | no | n/a | WCS | 14 | no | no | 0 | 3 | 16.7 | no | 0 | yes | no |
| 4 | 5 | FUP | Immature | yes | Invasive cervical resorption | Female | 14.4 | 32.2 | 17.7 | R2/4 | Included | 48 | 46 | Pristine ridge | no | 60 | Suture | 9 | no | no | 0 | 3 | 0.0 | no | 0 | no | no |
| 25 | 28 | FUP | Immature | yes | Infraposition | Female | 16.7 | 25.8 | 9.0 | R1/4 | Included | 28 | 35 | Pristine ridge | no | 90 | TTS | 26 | no | no | 0 | 5 | 50.0 | no | 0 | yes | no |
| 25 | 29 | FUP | Immature | yes | Infraposition | Female | 17.8 | 25.8 | 7.9 | R1/4 | Included | 18 | 45 | Pristine ridge | no | 120 | TTS | 78 | no | no | 0 | 3 | 16.7 | no | 0 | yes | no |
| 7 | 8 | FUP | Mature | yes | Success | Female | 28.7 | 38.4 | 9.8 | Ac | Included | 18 | 16 | Pristine ridge | yes | 110 | TTS | 26 | no | no | 1 | 3 | 16.7 | no | 0 | no | no |
| 8 | 9 | FUP | Mature | yes | Success | Female | 22.6 | 29.3 | 6.7 | Ac | Included | 28 | 45 | Pristine ridge | yes | 150 | TTS | 95 | no | no | 1 | 3 | 0.0 | no | 1 | no | no |
| 9 | 10 | FUP | Mature | yes | Success | Female | 12.2 | 22.9 | 10.8 | Ac | Dystopic | 33 | 33 | Pristine ridge | no | 90 | TTS | 34 | no | no | 0 | 3 | 16.7 | yes | 0 | no | no |
| 11 | 12 | FUP | Mature | yes | Success | Female | 19.0 | 27.0 | 7.9 | Ac | Included | 34 | 34 | Pristine ridge | yes | 150 | OA | 34 | no | no | 3 | 3 | 0.0 | yes | 1 | no | no |
| 14 | 16 | FUP | Mature | yes | Success | Female | 12.2 | 18.3 | 6.1 | Ac | Erupted | 25 | 35 | Pristine ridge | yes | 55 | TTS | 70 | no | no | 0 | 3 | 0.0 | no | 0 | no | no |
| 33 | 37 | FUP | Mature | yes | Root canal treatment | Female | 14.7 | 21.5 | 6.8 | Ac | Dystopic | 15 | 45 | Pristine ridge | yes | 75 | TTS | 211 | yes | no | 1 | 6 | 16.7 | no | 0 | no | Composite |
| 15 | 17 | FUP | Mature | yes | Root canal treatment | Female | 14.2 | 15.8 | 1.6 | Ac | Partially erupted | 23 | 43 | Pristine ridge | no | 120 | TTS | 39 | no | no | 0 | 3 | 16.7 | no | 0 | no | Composite |
| 19 | 21 | FUP | Mature | yes | Root canal treatment | Female | 20.1 | 26.3 | 6.2 | Ac | Partially erupted | 18 | 46 | Pristine ridge | no | 80 | TTS | 36 | no | no | 1 | 3 | 0.0 | no | 0 | no | Composite |
| 27 | 31 | FUP | Mature | yes | Replacement root resorption | Female | 13.3 | 16.6 | 3.3 | Ac | Partially erupted | 35 | 45 | Pristine ridge | no | 90 | TTS | 68 | no | no | 0 | 3 | 50.0 | no | 0 | yes | no |
| 1 | 1 | FUP | Mature | yes | Apicomarginal lesion | Male | 28.6 | 35.1 | 6.5 | Ac | Included | 13 | 13 | Pristine ridge | yes | 120 | OA | 163 | no | yes | 1 | 6 | 50 | no | 0 | no | no |
| 29 | 33 | FUP | Mature | yes | Apical  periodontitis | Female | 17.3 | 26.5 | 9.1 | Ac | Dystopic | 23 | 23 | Pristine ridge | yes | 60 | OA | 121 | no | no | 0 | 2 | 0.0 | no | 0 | no | no |
|  |  |  |  |  |  |  |  |  |  |  |  |  |  |  |  |  |  |  |  |  |  |  |  |  |  |  |  |
| 34 | 38 | PI | Immature | yes | n/a | Female | 18.2 | 22.9 | 4.7 | R3/4 | Included | 18 | 16 | Pristine ridge | no | 120 | TTS | 124 | no | n/a | n/a | n/a | n/a | n/a | n/a | n/a | n/a |
| 34 | 39 | PI | Immature | yes | n/a | Female | 15.3 | 22.9 | 7.7 | R2/4 | Included | 38 | 36 | Extraction socket | no | 90 | TTS | 119 | no | n/a | n/a | n/a | n/a | n/a | n/a | n/a | n/a |
| 35 | 40 | PI | Immature | yes | n/a | Female | 14.9 | 20.1 | 5.1 | R2/4 | Included | 28 | 45 | Pristine ridge | yes | 90 | Suture | 42 | no | n/a | n/a | n/a | n/a | n/a | n/a | n/a | n/a |
| 36 | 41 | PI | Immature | yes | n/a | Male | 11.8 | 23.0 | 11.2 | R2/4 | Erupted | 24 | 45 | Pristine ridge | no | 90 | TTS | 51 | no | n/a | n/a | n/a | n/a | n/a | n/a | n/a | n/a |
| 38 | 45 | PI | Immature | yes | n/a | Male | 14.3 | 21.1 | 6.8 | R2/4 | Dystopic | 43 | 45 | Pristine ridge | no | 300 | TTS | 37 | no | n/a | n/a | n/a | n/a | n/a | n/a | n/a | n/a |
| 40 | 47 | PI | Immature | yes | n/a | Male | 12.2 | 28.8 | 16.7 | R2/4 | Included | 45 | 35 | Pristine ridge | no | 60 | TTS | 58 | no | n/a | n/a | n/a | n/a | n/a | n/a | n/a | n/a |
| 41 | 48 | PI | Immature | yes | n/a | Male | 16.5 | 27.1 | 10.6 | R2/4 | Included | 28 | 46 | Extraction socket | yes | 90 | TTS | 51 | no | n/a | n/a | n/a | n/a | n/a | n/a | n/a | n/a |
| 42 | 49 | PI | Immature | yes | n/a | Male | 13.9 | 18.3 | 4.3 | R1/4 | Dystopic | 35 | 35 | Pristine ridge | no | 100 | TTS | 36 | no | n/a | n/a | n/a | n/a | n/a | n/a | n/a | n/a |
| 43 | 50 | PI | Immature | yes | n/a | Male | 17.5 | 24.0 | 6.5 | R3/4 | Dystopic | 35 | 35 | Pristine ridge | no | 135 | TTS | 101 | no | n/a | n/a | n/a | n/a | n/a | n/a | n/a | n/a |
| 37 | 43 | PI | Mature | yes | n/a | Female | 12.8 | 16.5 | 3.6 | Ac | Dystopic | 15 | 45 | Pristine ridge | no | 90 | TTS | n/a | no | n/a | n/a | n/a | n/a | n/a | n/a | n/a | n/a |
| 37 | 44 | PI | Mature | yes | n/a | Female | 12.8 | 16.5 | 3.6 | Ac | Included | 25 | 35 | Pristine ridge | no | 90 | TTS | n/a | no | n/a | n/a | n/a | n/a | n/a | n/a | n/a | n/a |
| 39 | 46 | PI | Mature | yes | n/a | Male | 14.8 | 22.8 | 7.9 | Ac | Dystopic | 45 | 45 | Pristine ridge | yes | 90 | TTS | 28 | no | n/a | n/a | n/a | n/a | n/a | n/a | n/a | n/a |
|  |  |  |  |  |  |  |  |  |  |  |  |  |  |  |  |  |  |  |  |  |  |  |  |  |  |  |  |
| 44 | 51 | PI | Immature | Loss | Inflammatory root resorption | Female | 12.2 | 20.6 | 8.3 | R2/4 | Erupted | 15 | 35 | Pristine ridge | no | 90 | TTS | 19 | n/a | n/a | n/a | n/a | n/a | n/a | n/a | n/a | n/a |
| 46 | 54 | PI | Immature | Loss | Invasive cervical resorption | Male | 12.6 | 16.7 | 4.1 | R3/4 | Erupted | 27 | 45 | Pristine ridge | no | 90 | TTS | 35 | n/a | n/a | n/a | n/a | n/a | n/a | n/a | n/a | n/a |
| 48 | 56 | PI | Immature | Loss | Replacement root resorption | Female | 13.3 | 19.4 | 6.1 | R1/4 | Dystopic | 45 | 45 | Pristine ridge | no | 120 | OA | 58 | n/a | n/a | n/a | n/a | n/a | n/a | n/a | n/a | n/a |
| 59 | 57 | PI | Immature | Loss | Apicomarginal lesion | Male | 16.7 | 20.8 | 4.1 | R2/4 | Partially erupted | 48 | 47 | Pristine ridge | yes | 60 | Suture | $ | n/a | n/a | n/a | n/a | n/a | n/a | n/a | n/a | n/a |
| 45 | 53 | PI | Mature | Loss | Replacement root resorption | Female | 15.0 | 17.9 | 2.9 | Ac | Partially erupted | 47 | 46 | Pristine ridge | no | 80 | TTS | 34 | n/a | n/a | n/a | n/a | n/a | n/a | n/a | n/a | n/a |
| 47 | 55 | PI | Mature | Loss | Replacement root resorption | Female | 10.3 | 17.3 | 6.9 | Ac | Partially erupted | 46 | 46 | Extraction socket | no | 130 | TTS | 36 | n/a | n/a | n/a | n/a | n/a | n/a | n/a | n/a | n/a |
| 50 | 58 | PI | Mature | Loss | Replacement root resorption | Male | 16.5 | 18.1 | 1.5 | Ac | Dystopic | 13 | 33 | Extraction socket | no | 180 | TTS | 42 | n/a | n/a | n/a | n/a | n/a | n/a | n/a | n/a | n/a |
| 50 | 59 | PI | Mature | Loss | Replacement root resorption | Male | 16.5 | 18.1 | 1.5 | Ac | Included | 43 | 43 | Extraction socket | no | 180 | TTS | 42 | n/a | n/a | n/a | n/a | n/a | n/a | n/a | n/a | n/a |
| 5 | 60 | FUP | Mature | Loss | Invasive cervical resorption | Male | 12.3 | 17.1 | 4.8 | Ac | Partially erupted | 35 | 14 | Extraction socket | no | 50 | WCS | 28 | n/a | n/a | n/a | n/a | n/a | n/a | n/a | n/a | n/a |

Supplementary Table 1 Summary of patient, site, surgical, and clinical characteristics across study groups and classifications. Abbreviations: FUP, follow-up examination; PI, phone inquiry; TTS, Titan-Trauma-Splint; WCS, wire-composite splint; OA, orthodontic appliance.
